# Uterine Affections

**Published:** 1828-11

**Authors:** 


### HOSPITAL REPORTS,

*(Principally condensed from various Periodical Publications.)*

### UTERINE AFFECTIONS.

### *Case of Prolapsus of the Uterus.*

July 31st.—A young woman, aged eighteen, six months before her admission into St. Thomas's Hospital, exerted herself in lifting another person out of a coach, in consequence of which prolapsus of the uterus took place, together with considerable hemorrhage. She fainted immediately, and was carried home; but in the course of a month was so much better as to be married. Since then she has had constant pain and tenderness over the region of the uterus; pain and extreme soreness at the umbilicus; and frequent vomiting. The uterus has made its appearance externally two or three times since the first occurrence of the procidentia, but has been replaced by a medical practitioner, who has also made use of some treatment to diminish the more urgent symptoms.

On making an examination, Dr. Elliotson discovered that the uterus was still in the vagina, forming a small tumor, the neck of which was tightly girt by the os uteri. She had pain and tenderness in the situations above described, with a tongue white at each side, and red in the centre and tip, and a quick and sharp pulse.

Dr. Elliotson thought that the uterus was in a state of inflammation, and that the gastric symptoms arose merely from the sympathy of the stomach with the former organ.

The patient had, a short time before her admission, suffered from inflammation of the throat, followed by ulceration.

**Hirudines xx. abdomini, hodie et cras.—Slops.**

August 4th.—The pain and tenderness, which had disappeared, having returned, and the stomach being again irritable, twenty leeches were applied.

5th.—Great pain and tenderness of lower part of abdomen. Pulse 120, and hard; pain at the pit of the stomach; vomiting.

Dr. Roots saw the patient this day, Dr. E. having left town.

**He ordered V.S. ad ℥xxj.—Ol. Ricini ℥ss. statim.—Hyd. Subm. gr. ij. Opii gr. ¼; Antim. Tart. gr. $\frac{1}{6}$, in pil. sextis horis.**

6th.—The bleeding removed all pain and tenderness of abdomen.

7th.—No pain. Stomach still irritable. In a few days all inflammatory symptoms had disappeared. The prolapsus continuing, an astringent injection was prescribed, and a pesary ordered to be worn. These partially relieved the displacement, and in a few days she left the hospital.

### *Polypus of the Uterus.*

July 31st.—A woman, aged forty-seven, in whom the menses had long stopped, began to feel severe pain at the lower part of the abdomen. A week after, when she was coughing, a small tumor came through the external orifice of the vagina, with a quantity of blood. This was three months before her admission into St. Thomas's Hospital. Since then she has been subject to violent shooting pains of hips, loins, and back; nausea; a discharge of fœtid yellow matter, and occasional flooding.

On examination, a polypus was found attached to the mouth and neck of the uterus. It was of the size of a small apple, and attached by a narrow base.

To remove it, Mr. Tyrrell put a needle, armed with a strong ligature, through the neck of the tumor, which he firmly tied with the same ligature: he then removed the greater part of the polypus with a bistoury, leaving that portion on which the ligature was still attached. Little blood was lost at the time; but so much hemorrhage occurred a few hours after, that the patient nearly sunk. It was at length checked by pressure and oleum terebinthinæ.

---

### *Chronic Inflammation of the Neck of the Uterus.*

The extirpation of the neck of the uterus has become latterly so common, that doubts have in consequence arisen as to its necessity in some of the cases in which it has been performed; and the anatomical examination of the parts so removed has shewn these doubts to be well founded. The following case will give additional strength to the reasonings which have been founded upon the resources of nature and art which some practitioners appear to be ignorant of, and which may prevent them from performing operations that have too often caused the death of the unhappy sufferers.

There was lately in La Charite a young woman, who had been ill about four years and a half. At that time, being in the fifth month of her pregnancy, she received a blow upon the abdomen, which in a few hours produced a miscarriage. Great affliction having made her indifferent to life, she took no care of herself, neglecting even the common precautions. The menses returned, but with great pain, and were very irregular; at the same time she experienced dull pains in the hypogastrium, loins, and groins, with an abundant discharge from the vagina, pain in the stomach, and continual headache. This condition lasted about a year; but, being obliged to work for her living, her occupation (that of washing) caused a continual pressure upon the abdomen, which augmented her disease, so that the pain at length became insupportable; and she was admitted into La Charité, under the care of M. Fouquier.

The above symptoms rendered the seat of the malady evident enough, and an organic lesion of the neck of the uterus was supposed to be the cause; but the nature of the affection was disputed. In the mean while, numerous leeches were applied to the pudenda, and even to the neck of the uterus itself. By these means she was speedily relieved; but the enlargement of the part was undiminished, as was also the acrid discharge from the vagina.

M. Roux was consulted. After an attentive examination, the disease was considered cancerous, an operation proposed, and eagerly accepted. Each day M. Roux examined the patient very attentively, and each day, notwithstanding the intreaties of the woman, he postponed the operation. At length it was decided upon. The patient was placed upon the table, and every thing was prepared, when the surgeon, who had actually laid hold of the neck of the uterus, and had the bistoury in his hand, again sent the patient to her bed. A severe peritonitis followed the attempts that had been made: the repeated application of leeches succeeded in subduing this disease, and in a few weeks the symptoms disappeared. The original disease was then again thought of, and was treated with antiphlogistics and emollients. By degrees the swelling of the neck of the uterus disappeared, the menses returned, the discharge ceased, and the patient was able to quit the hospital, (where she had been upwards of ten months,) regarding herself as cured. A year elapsed without her experiencing any return of the disease: she was able to pursue a tolerably active employment the whole of that time; and, in fact, excepting some occasional attacks of indigestion, her health was very good.

About the month of November last, having suffered a severe disappointment, she was suddenly seized with the same symptoms she had before experienced; but, until January, she sought for no relief. On the 30th of that month she was received into the hospital. Her sufferings were at their height: the pudenda were irritated by a yellowish discharge, which ulcerated the neighbouring parts; the neck of the uterus formed a flattened tumor, red, very sensible, and covered with inequalities of a scirrhous hardness, and presenting in front an ulceration of an irregular form, about the size of a thirty-sous piece. The abdomen was swollen in the hypogastric region, but without pain in that part. Leeches, warm baths, semicupic fomentations, and soothing measures of all kinds, were made use of. A marked amendment was the consequence: the neck of the uterus became progressively softer, losing its increased size and sensibility. These changes followed the use of fomentations injected by the vagina. The belly, nevertheless, continued swollen, and the patient proved to be with child; in consequence of which she left the hospital.

This premature departure is to be lamented, because, although far advanced, the cure was not complete. It is, nevertheless, quite certain that this woman had not a cancerous affection of the neck of the uterus, and that, if she had fallen into other hands than those of M. Roux, she would have undergone an untimely, perhaps a fatal, operation.

---

*Rupture of the Uterus at the Time of Quickening.*

Mrs. ——, æt. twenty, lost her life under the following circumstances: She had been married about fifteen months, and, until the time of her conception, had enjoyed tolerable health; but since that period had suffered considerably from deep-seated pain in the back and uterine region, together with other symptoms threatening abortion.

Before her marriage, and up to the time of conception, she had experienced an unusual degree of pain at each menstrual period; and the catamenial discharge was exceedingly scanty. Her death appeared in some measure accelerated by an excursion to Greenwich, in company with her husband, as shortly after her arrival there she was attacked with vomiting and syncope, and in less than an hour she ceased to exist.

Upon examination, it was discovered that a rent of about five inches in length had taken place in the uterus, extending itself from the cervix upwards at its anterior part, and rupturing a portion of the placenta. The fœtus lay in front of the uterus, enveloped by its internal membrane, and surrounded by coagulated blood, a quantity of which was also found between the intestines and in the cavity of the pelvis. The uterus itself was covered with dark-coloured spots, and easily lacerable. The ovaries were also in a state of disease: the one containing hydatids, the other with the same dark-coloured spots as the uterus. The fœtus appeared healthy, and is supposed by its movements to have caused the rupture of the uterus.

The above case is related by Mr. Else, in the Medical Gazette.

---

EXTIRPATION OF THE UTERUS.

